# Nationwide survey on current treatments for IgA nephropathy in Japan

**DOI:** 10.1007/s10157-013-0779-7

**Published:** 2013-03-22

**Authors:** Keiichi Matsuzaki, Yusuke Suzuki, Junichiro Nakata, Naoko Sakamoto, Satoshi Horikoshi, Tetsuya Kawamura, Seiichi Matsuo, Yasuhiko Tomino

**Affiliations:** 1 Division of Nephrology, Department of Internal Medicine, Juntendo University Faculty of Medicine, 2-1-1 Hongo, Bunkyo-ku, Tokyo, 113-8421 Japan; 2 National Research Institute for Child Health & Development, Tokyo, Japan; 3 Jikei University School of Medicine, Tokyo, Japan; 4 Nagoya University Graduate School of Medicine, Nagoya, Aichi Japan; 5 Progressive Renal Diseases Research, Research on Intractable Disease, from the Ministry of Health, Labour and Welfare of Japan, Japan, http://jin-shogai.jp/mt/public/hp/

**Keywords:** IgA nephropathy, Nationwide survey, Questionnaire survey, Tonsillectomy and steroid pulse therapy

## Abstract

**Background:**

A wide variety of treatments, including tonsillectomy and steroid pulse therapy (TSP), are performed for the various stages of IgA nephropathy (IgAN) in Japan. However, the current status of treatments for IgAN patients in Japan is still unclear. The objective of the present study was to investigate the current status of treatments for IgAN patients.

**Methods:**

A nationwide survey was conducted in 2008 by sending questionnaires to the 1,194 teaching hospitals of the Japanese Society of Nephrology (JSN) via Progressive Renal Diseases Research, Research on intractable disease, from the Ministry of Health, Labour and Welfare of Japan.

**Results:**

Among the total 376 hospitals (31.4 %) that responded, 188 hospitals (66.2 % in the internal medicine departments) performed TSP, out of which 137 hospitals (61.4 %) had begun to perform TSP in the period from 2004 to 2008. The following two major steroid pulse protocols in TSP were used: (1) three cycles over 3 consecutive weeks and (2) three cycles every 2 months. Approximately 68 % of pediatric hospitals (68 hospitals) performed combination therapy with prednisolone, azathioprine, heparin-warfarin and dipyridamole. The clinical remission rates for hematuria and proteinuria after TSP tended to be higher than those following other corticosteroid therapies. Almost all hospitals prescribed antiplatelet agents and renin angiotensin system inhibitor (RAS-I).

**Conclusion:**

In addition to popular treatments such as antiplatelet agents and RAS-I, TSP is becoming a standard treatment for adult IgAN patients in Japan.

## Introduction

IgA nephropathy (IgAN) is the most common primary chronic glomerulonephritis in the world, and is recognized as one of the major causes of end-stage kidney disease (ESKD) [1–5]. Although IgAN was initially believed to represent a benign condition, recent studies [6] have shown that 30–40 % of patients progress to ESKD within 10–25 years from its apparent onset. Therefore, treatment strategies to decrease the risk of IgAN progressing to ESKD would have substantial health benefits [7]. However, disease-specific therapy for IgAN patients has not been established because the pathogenesis of IgAN is still a matter of debate.

As annual check-ups including urinalysis are well established in Japan, patients in various stages of IgAN can be managed and are provided a wide variety of treatments. Oral corticosteroid, steroid pulse therapy, tonsillectomy and steroid pulse therapy (TSP), antihypertensive agents, immunosuppressants, antiplatelet agents and anticoagulants are listed in the regional guidelines of Japan [8].

Corticosteroid therapy is now a popular treatment for IgAN patients after being first reported by Kobayashi [9]. Although the clinical value of intravenous steroid pulse therapy was demonstrated by Pozzi et al [10], no consensus exists for the corticosteroid dose and administration route (oral or intravenous infusion). TSP has recently become a popular standard treatment in Japan. However, the current status of IgAN treatment in Japan is still unclear because no nationwide study has been conducted. Thus, we conducted a nationwide survey using a questionnaire through the Progressive Renal Diseases Research, Research on intractable disease, from the Ministry of Health, Labour and Welfare of Japan.

## Methods

We sent questionnaires by mail to 1,194 hospitals (Internal Medicine, 803; Pediatrics, 391), which are teaching hospitals in the Japanese Society of Nephrology (JSN), between October 30 and December 27 in 2008. The questionnaire covered treatment details provided for IgAN and their outcomes (Table 1).

**Table 1:**
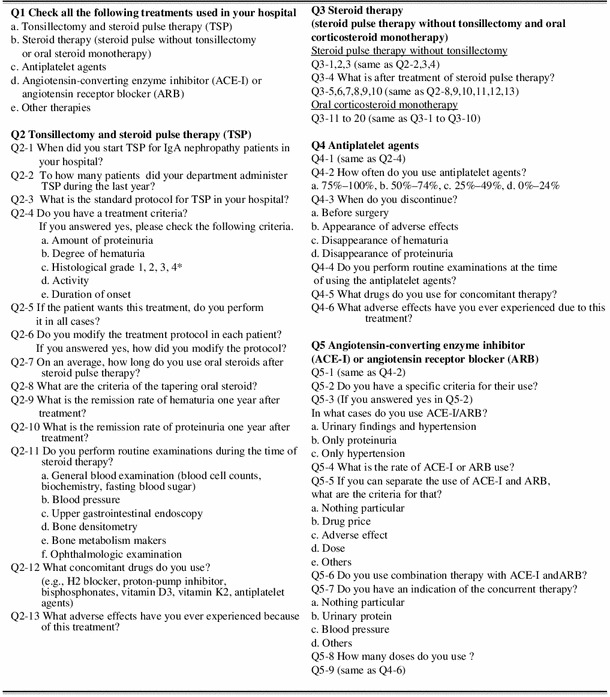
Questionnaire

*1* good prognosis group, *2* relatively good prognosis group, *3* relatively poor prognosis group, *4* poor prognosis group

*Criteria for histological grading from IgA nephropathy (IgAN) clinical guidelines in Japan

## Results

A total of 376 hospitals (31.4 %) (Internal Medicine 284; Pediatrics 92) responded. The mean number of beds in these hospitals was 581.

### Tonsillectomy and steroid pulse therapy (TSP)

**Fig. 1:**
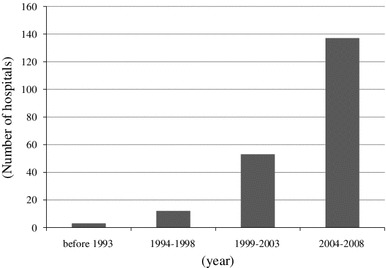
Starting year for tonsillectomy and steroid pulse therapy (TSP). TSP spread rapidly in Japan from 2004 to 2008

**Fig. 2:**
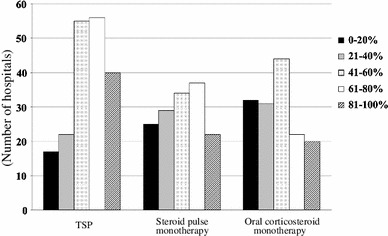
Clinical remission rate for hematuria based on treatment. The clinical remission rate for hematuria in many hospitals using TSP was higher than that after steroid pulse without tonsillectomy or oral corticosteroid monotherapy

**Fig. 3:**
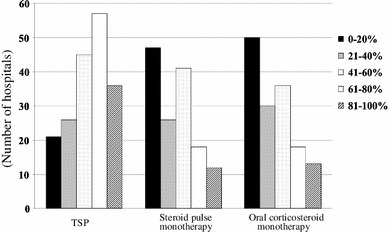
Clinical remission rate of proteinuria based on the treatment. The clinical remission rate for proteinuria using TSP was higher than that using steroid pulse without tonsillectomy or oral corticosteroid monotherapy

A total of 188 internal medicine hospitals (66.2 %) stated that they had performed TSP. Steroid pulse therapy was always combined with tonsillectomy in 72 (38.3 %) hospitals. The starting year for TSP is shown in Fig. 1. The annual number of patients who received TSP treatment ranged widely from 1 to 200 patients per year. Steroid pulse therapy using 500–1,000 mg/day (or 20–30 mg/kg/day) methylprednisolone (m-PSL) was performed using the following two major protocols; (1) three times over 3 consecutive weeks (47.8 %), and (2) three times every 2 months (18.9 %). The number of steroid pulses varied at each hospital (24 hospitals, once; 12 hospitals, twice; 92 hospitals, three times). In total, 179 hospitals (80.2 %) did not change the protocol for each patient. Almost all facilities prescribed oral prednisolone after the steroid pulse therapy. A total of 141 hospitals (63.2 %) had criteria for tapering oral prednisolone. The most cited indication for the therapy was the histological findings (164 hospitals, 87.2 %), and other indications were proteinuria grade (156 hospitals, 83.0 %), disease activity (104 hospitals, 55.3 %), hematuria grade (56 hospitals, 29.8 %) and duration from onset (38 hospitals, 20.2 %). In addition, 109 hospitals (48.9 %) performed TSP if the patients wanted and the doctors judged to need the treatment. Figures 2 and 3 show the clinical remission rates for hematuria and proteinuria. The most frequent remission rate ranged from 60 to 80 %. Table 3 shows the routine examination before TSP, concomitant drugs and adverse effects.

### Steroid pulse therapy without tonsillectomy

A total of 192 hospitals (51.1 %) performed steroid pulse therapy without tonsillectomy (Table 2). Most of the hospitals (183 hospitals, 95.3 %) performed steroid pulse therapy for less than 10 patients annually. Only six hospitals performed steroid pulse therapy for more than 11 patients per year. The main protocol of steroid pulse therapy was 500–1,000 mg/day m-PSL for 3 consecutive days. The number of times steroid pulses were varied among hospitals (34 hospitals, once; 31 hospitals twice; 65 hospitals, three times). The most cited indication for this therapy was histological findings and proteinuria grade (137 hospitals, 71.4 %), and other indications were disease activity (97 hospitals, 50.5 %), hematuria grade (30 hospitals; 15.6 %) and duration from onset (22 hospitals, 11.5 %). All hospitals prescribed oral prednisolone after the steroid pulse therapy. In total, 102 hospitals (53.1 %) had criteria for tapering oral prednisolone. Although the clinical remission rate for hematuria ranged between 60 and 80 % (Fig. 2), the remission rate for proteinuria was ranged between 0 and 20 % (Fig. 3). Table 3 shows the routine examinations performed before the steroid pulse therapy without tonsillectomy, concomitant drugs and adverse effects.

**Table 2:** Number of hospitals for each treatment

	Total (%) *n* = 376	Internal medicine (%) *n* = 284	Pediatrics (%) *n* = 92
TSP	223 (59.3)	188 (66.2)	35 (38.0)
Steroid pulse monotherapy	192 (51.1)	159 (56.0)	33 (35.9)
Oral corticosteroid monotherapy^a^	184 (48.9)	156 (54.9)	28 (30.4)
Antiplatelet agents	351 (93.4)	275 (96.8)	76 (82.6)
RAS-I	371 (98.7)	283 (99.6)	88 (95.7)

*TSP* tonsillectomy and steroid pulse therapy, *RAS*-*I* renin–angiotensin system inhibitor

^a^Including combination therapy (prednisolone, azathioprine, heparin-warfarin, and dipyridamole)

**Table 3:** Routine examinations, concomitant drugs, and adverse effects for each treatment

	Routine examination (hospitals, %)	Concomitant drugs (hospitals, %)	Adverse effects (hospitals, %)
TSP	General blood examination (221, 99.1), Blood pressure (202, 90.6), Ophthalmologic examination (108, 48.4), Bone densitometry (107, 48.0), Upper gastrointestinal endoscopy (40, 17.9), Bone metabolism maker (20, 9.0)	H2 blocker or proton-pump inhibitor (207, 92.8), Antiplatelet agent (157, 70.4), Vitamin D3 (91, 40.8), Vitamin K2 (15, 6.7)	Steroid-induced diabetes (32, 14.3), Steroid-induced psychosis (17, 7.6), Moon face (12, 5.4), Steroid osteoporosis (6, 2.7), Postoperative pain (6, 2.7), Bleeding (5, 2.2), Loss of taste (3, 1.3)
Steroid pulse monotherapy	General blood examination (147, 76.6), Blood pressure (135, 70.3), Ophthalmologic examination (75, 39.0), Bone densitometry (74, 38.5), Upper gastrointestinal endoscopy (28, 14.6), Bone metabolism maker (16, 8.3)	H2 blocker or proton-pump inhibitor (137, 71.4), Antiplatelet agent (22, 11.5), Vitamin K2 (13, 6.8)	Steroid-induced diabetes (13, 6.8), Steroid-induced cataract (7, 3.6), Pneumonia (5, 2.6), Moon face (4, 2.1), Central obesity (4, 2.1)
Oral corticosteroid monotherapy*	General blood examination (128, 69.6), Blood pressure (116, 63.0), Bone densitometry (56, 30.4), Ophthalmologic examination (55, 29.9), Upper gastrointestinal endoscopy (20, 10.9), Bone metabolism maker (15, 8.2)	H2 blockers or proton-pump inhibitors (111, 60.3), bisphosphonates (74, 40.2), Vitamin D3 (56, 30.4), Antiplatelet agents (26, 14.1), Vitamin K2 (9, 4.9)	Steroid-induced diabetes (11, 6.0), Steroid-induced cataract (5, 2.7), Steroid-induced psychosis (4, 2.1), Moon face (3, 1.6), Steroid-induced osteoporosis (3, 1.6)

*Including combination therapy (prednisolone, azathioprine, heparin-warfarin, and dipyridamole) TSP, tonsillectomy and steroid pulse therapy

### Oral corticosteroid monotherapy (including combination therapy)

A total of 184 hospitals (48.9 %) performed oral corticosteroid monotherapy (Table 2). Most of the hospitals (149, 81.0 %) performed this therapy for less than 10 patients annually, and only 10 hospitals performed it for more than 11 patients. The most frequent initial dose in the internal medicine and pediatric departments was 21–40 and 1–2 mg/kg/day, respectively. The most frequent duration of medication was 24 months (54 hospitals, 28.7 %), and the duration of medication varied in each hospital. Seventy-four hospitals (40.2 %) had tapering criteria, and 68 hospitals (68.5  % in pediatric hospitals) provided a combination therapy of prednisolone, azathioprine, heparin-warfarin and dipyridamole. The most cited indication for this therapy was the proteinuria grade (140 hospitals; 76.1 %). Other indications included histological findings (129 hospitals, 70.1 %), disease activity (93 hospitals, 50.5 %), hematuria grade (31 hospitals, 16.8 %) and duration from onset (19 hospitals, 10.3 %). The most frequent clinical remission rate of hematuria was 40–60 % (Fig. 2), and that of proteinuria was 0–20 % (Fig. 3). Table 3 shows the routine examinations performed before oral corticosteroid monotherapy, concomitant drugs and adverse effects.

### Antiplatelet agents

**Fig. 4:**
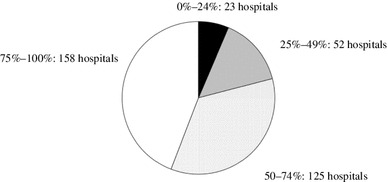
Prescription rate for antiplatelet agents in each hospital. Almost 40 % of the hospitals prescribed for 75–100 % patients in their hospital

A total of 351 hospitals (93.4 %) prescribed antiplatelet agents (Table 2). The majority of hospitals (188; 53.6 %) prescribed the antiplatelet agents in all cases. The prescription rate in each hospital is shown in Fig. 4. The main reason for discontinuation was scheduled surgery (313 hospitals, 89.3 %). The routine examination before this treatment was mainly a general blood examination. Major adverse effects were headache and gastrointestinal symptoms.

### Renin-angiotensin system inhibitor (RAS-I)

**Fig. 5:**
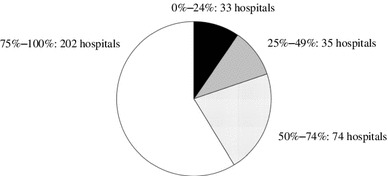
Prescription rate for renin-angiotensin system inhibitors in each hospital. More than 50 % hospitals prescribed for 75–100 % patients in each hospital

A total of 371 hospitals (98.7 %) prescribed RAS-I (Table 2), but 226 hospitals (60.1 %) did not have criteria for this treatment. The prescription rate is shown in Fig. 5. Most hospitals did not have clear criteria for the choice between angiotensin-converting enzyme inhibitor (ACE-I) and angiotensin receptor blocker (ARB), and 218 hospitals (58.8 %) prescribed concurrently ACE-I and ARB. The most indicated criteria for the combination was proteinuria (160 hospitals, 73.4 %) and blood pressure (94 hospitals, 43.1 %). Adverse effects include hyperkalemia, elevation of serum creatinine, hypotension, dizziness and dry cough.

## Discussion

A wide variety of treatments for IgAN exist in Japan because various stages of disease can be observed and managed. The current treatment situation has been unclear until now because no nationwide study has been conducted regarding IgAN treatment. The present study assessed the precise situation of treatment for IgAN in Japan.

TSP was first reported by Hotta et al. [11] in 2001. Many clinical studies on TSP have been reported from Japan since 2001 [12–14]. Miura et al. conducted a multicenter retrospective cohort study, and reported that TSP was effective for patients with early-stage IgAN if performed within 5 years of onset and for those who have daily proteinuria <1.1 g and serum creatinine <1.5 mg/dl [15]. However, the details of TSP (protocols, indication, clinical remission rate, etc.) varied in each report, and the current TSP situation was thus unclear. Our results show that almost 70 % of internal medicine hospitals performed TSP. Almost 40 % of hospitals always added combined steroid pulse therapy with tonsillectomy. Moreover, almost 60 % hospitals began TSP in the period between 2004 and 2008 (Fig. 1), indicating that TSP spread through Japan quickly and has become the major therapeutic approach for IgAN in the last decade. We also observed that the clinical remission rates for both hematuria and proteinuria following TSP tended to be higher than those resulting from steroid pulse without tonsillectomy or oral corticosteroid monotherapy (Figs. 2, 3). This may be one of the main reasons for the quick spread of this therapy in Japan. In previous reports, TSP protocols have varied. In particular, the number of steroid pulses given during TSP varied in each report [11–13]. Our results showed that there are two major protocols for TSP in Japan. One is a protocol in which the steroid pulses are administrated three times, with a steroid pulse every week, on the basis of the original report by Hotta et al. [11]. Another is in which steroid pulses are administrated three times every 2 months, based on previous report by Pozzi et al. [10]. We did not find a clear difference in clinical efficacy between two methods.

The Japanese Pediatric IgA Nephropathy Treatment Study Group advocated combination therapy for childhood IgAN in their 2008 guideline [16]. A number of studies by Japanese groups [17–19] have reported beneficial outcomes in childhood IgAN using the combination therapy with prednisolone, azathioprine, heparin-warfarin and dipyridamole. The rationale for this treatment is as follows; (1) corticosteroids and immunosuppressive agents reduce serum IgA production and minimize the abnormal immune response and inflammatory events following glomerular IgA deposition, and (2) heparin-warfarin and dipyridamole are used to inhibit the mediators of glomerular damage [17]. Our results demonstrated that 68 hospitals (68.5 % of pediatric hospitals) performed the combination therapy, suggesting that combination therapy is a standard therapy for pediatric IgAN in Japan. Pozzi et al. [20] recently demonstrated that clinical outcomes in adults are not different between treatment with corticosteroids alone and corticosteroids with oral azathioprine. In contrast, Kamei et al. [21] reported that the combination therapy improves the long-term outcome in childhood IgAN. Because these two studies enrolled different populations, this difference may provide a clue of the indications for this treatment.

A recent meta-analysis showed that the use of corticosteroids IgAN patients is associated with a significant decrease in the risk of ESKD and urinary protein excretion [22]. Although the practice pattern (with or without tonsillectomy, immunosuppressants, etc) was not standardized, almost 70 % hospitals were found to perform corticosteroid therapy. Consequently, one can conclude that corticosteroid therapy has become standard in Japan. In particular, TSP and combination therapy are popular in internal medicine and pediatric departments, respectively.

Intraglomerular coagulation, either through local activation of blood coagulation or impaired removal by the fibrinolytic system, has been proposed as one of the factors causing glomerular injury in IgAN [23]. Previous studies including meta-analysis [24–27] reported beneficial effects of anti-platelet agents for IgAN. Therefore, antiplatelet agents are listed in the Japanese regional guidelines [8]. In fact, the national health insurance covers dipyridamole for glomerulonephritis and dilazep hydrochloride for IgAN. On the contrary, Fleoge et al. [28] did not recommend using antiplatelet agents in patients with IgAN because most studies on antiplatelet agents are often combined with immunosuppressants and were retrospective and nonrandomized. Moreover, the Kidney Disease: Improving Global Outcomes (KDIGO) Clinical Practice Guideline for Glomerulonephritis concluded that there is no benefit for antiplatelet agents alone because patients received other concomitant therapies in Japanese studies [29]. Our results suggest that almost all Japanese hospitals (351, 93.4 %) prescribed antiplatelet agents for IgAN. It is thought that Japanese nephrologists prescribe these drugs based on previous studies and for compliance with regional guidelines. In future, we need to confirm the effects of antiplatelet agents in a large cohort study from Japan.

RAS-I is effective for glomerular hypertension, podocyte injury and tubulointerstitial injury, and thus is prescribed for glomerulonephritis. Amelioration of glomerular injury and fibrosis by ARB has been demonstrated in animal models of IgAN [30]. Because several studies, including randomized controlled trials [31–33], have reported the effectiveness of RAS-I for IgAN, recent guidelines [29] recommend this therapy for IgAN. Tomino et al. [34] and Moriyama et al. [35] reported the beneficial effects of IgAN in Japan. Furthermore, our results revealed that almost all hospitals (371, 98.7 %) prescribed RAS-I for IgAN, indicating that RAS-I is a popular treatment in Japan. The combination of ACE-I and ARB has antiproteinuric effects greater than monotherapy in normotensive IgAN [36]. The present study revealed that 218 hospitals (58.8 %) prescribed ACE-I and ARB concurrently. The indications for concurrent use are proteinuria and blood pressure, suggesting that they aim to renoprotect through antiproteinuric effects.

Our study has several limitations. First, there was a possibility of selection bias. The response rate was only 31.4 % of 1,194 hospitals. Those hospitals tended to be large hospitals; thus, our results could not reflect the treatments in small hospitals and clinics. Second, a possibility for measurement bias regarding clinical efficacy existed. Because we did not strictly define “clinical remission” in this study, treatment efficacy depended on the judgment of each hospital. Third, the questionnaire asked about all treatments in each hospital; thus, we could not analyze and estimate the priority of the treatments. Fourth, the questionnaire surveyed all IgAN stages, but it is well known that IgAN has a heterogeneous disease course; therefore, treatments may depend on stage. In future, we need to conduct an investigation of the treatments for each stage of IgAN.

In conclusion, corticosteroid therapy, along with antiplatelet agents and RAS-I therapy, has become a standard treatment for IgAN in Japan. Although we observed that the corticosteroid therapy protocol varied, TSP is becoming a standard treatment, at least for adult IgAN. Further studies are required to compare the efficacy of each treatment and to determine the standard therapy for each stage of IgAN.
